# Physiotherapy Intervention for Promoting Comfort in Palliative Care Patients: A Focus Group Study

**DOI:** 10.3390/cancers17132167

**Published:** 2025-06-27

**Authors:** Daniela Filipa dos Santos Domingos, Ana Querido, Vanda Varela Pedrosa

**Affiliations:** 1 School of Health Sciences, Polytechnic Institute of Leiria, 2411-901 Leiria, Portugal; 5230011@my.ipleiria.pt; 2 School of Health Sciences, Polytechnic Institute of Leiria, Center for Innovative Care and Health Technology (ciTechCare), 2414-016 Leiria, Portugal; ana.querido@ipleiria.pt; 3 RISE Health Research Group, University of Porto, 4200-450 Porto, Portugal

**Keywords:** physiotherapy, palliative care, comfort, multidisciplinary team integration

## Abstract

**Background/Objectives**: Population aging and the rise in chronic diseases challenge healthcare systems to adopt person-centered approaches, especially in palliative care (PC), where symptom management remains limited. Physiotherapy plays a key role in alleviating discomfort but faces inconsistent integration in Portugal due to lack of recognition. Variations in intervention methods hinder uniform care delivery, limiting timely patient access to comfort-focused treatments and knowledge. This study aims to deepen the understanding of physiotherapy’s role in Portuguese PC to improve its integration into teams and enhance patient access to comfort care. **Methods**: This study used a descriptive qualitative approach with online focus groups (FG), guided by Krueger and Casey’s methodology and adhering to the COREQ checklist. A non-probabilistic convenience sample of physiotherapists working in palliative care across mainland Portugal and the islands was selected based on inclusion criteria. Three FGs were planned with up to ten participants each. However, due to availability and attendance issues, only 15 physiotherapists participated: 5 in FG1 (in-hospital PC units), 6 in FG2 (inpatient units), and 4 in FG3, the minimum appropriate number from community-based units. **Results**: Physiotherapy plays a crucial yet underrecognized role in PC, emphasizing the need for its full integration into care teams rather than reliance on late, on-call referrals. Techniques such as positioning, mobilization, pain and dyspnea relief, adapted exercises, massage, music therapy, and emotional support are employed. Conventional physiotherapy tools are used and personalized according to the patient’s context, duration, setting, dosage, and individual needs. **Conclusions**: Physiotherapy should be recognized as a fundamental part of PC, contributing not only to the prolongation of life but also to ensuring comfort and dignity for patients and their families. To achieve this, its role within multidisciplinary teams must be strengthened and supported by regulations that guarantee access and the formal integration of physiotherapists. However, a significant gap remains in patients’ regular access to comfort-focused interventions at the appropriate time, perhaps due to the considerable variation in physiotherapy practices depending on the patient and care setting, which presents a challenge for knowledge development both in Portugal and globally.

## Simple Summary

The increase in average life expectancy presents significant challenges for healthcare services, particularly in palliative care, where the early identification of diverse needs, most notably comfort, can substantially improve quality of life. In this context, the aim of this qualitative study, based on three online focus groups, is to explore the potential value that physiotherapy may offer within these services. Findings confirmed that, regardless of the type of palliative care setting, physiotherapy plays a crucial role in effectively enhancing the well-being and comfort of patients and their families. This is achieved through positioning and mobilization techniques, pain and dyspnea relief, adapted therapeutic exercise, massage, music therapy, and emotional support. This study also highlighted the need for physiotherapy to be fully and equitably integrated into interdisciplinary teams and emphasized the urgency of regulatory measures to ensure access to physiotherapy across all regions of the country within the context of palliative care.

## 1. Introduction

Scientific and technological advances have increased life expectancy and aging, raising the occurrence of chronic conditions and disabilities. This trend challenges healthcare systems to adopt person-centered approaches, particularly in end-of-life care with limited symptom management [1,2,3,4,5,6].

Palliative care (PC) is essential to ensuring quality of life and dignity in advanced illness, both globally and in Portugal. Non-pharmacological interventions, especially those delivered by physiotherapists, are increasingly recognized for relieving discomfort, improving function, and supporting patients and families through an interdisciplinary, humanized approach addressing physical, psychological, social, and spiritual needs [3,7,8,9,10,11,12,13]. Comfort-focused care is especially valuable for individuals facing incurable, progressive illnesses with life-threatening prognoses. Comfort is understood as a holistic response to needs, whether active, passive, or collaborative, and includes three types: relief, ease, and transcendence. These are experienced across four dimensions: physical, psychosocial, environmental, and spiritual, fostering well-being. In specialized PC, this multidimensional comfort offers patients and families meaningful support, as emphasized by Boudiab and Kolcaba and others [14,15,16,17].

In mainland Portugal, specialized PC teams within the National Health Service (SNS) are part of the National Palliative Care Network, including In-Hospital Care Units, Inpatient Palliative Units, and Community Palliative Care Support Teams [11,18]. In the autonomous regions, organization differs; Madeira has a Regional Palliative Care Network [19], while the Azores integrate PC into the Regional Integrated Continuing Care Network [20].

In mainland Portugal and the autonomous regions, in-hospital PC units provide care for patients with advanced or acute stages of serious illnesses, including cancer, neurological disorders, chronic respiratory conditions, renal failure, or multiple organ failure. These units offer continuous monitoring and longer-term interventions to improve quality of life throughout the disease stages, including end-of-life care [9,21,22]. Physiotherapists typically begin within general hospital care before joining palliative teams, focusing on symptom relief and mobility through personalized care, early prevention, symptom management, and close collaboration with physicians, despite time and resource limitations [21,22,23].

Palliative care units deliver care within a single institution to individuals with incurable or distressing illnesses, aiming to ensure the best possible quality of life regardless of prognosis. Physiotherapy is flexibly integrated to provide function-focused, individualized care within interdisciplinary teams, including mobilization, positioning, therapeutic exercises, symptom relief, and psychosocial support. Barriers include delayed referrals and a shortage of trained professionals. Key strategies involve training, tailored care, and caregiver involvement post-discharge [21,22,23].

Community-based units provide care at home, supporting patients in familiar settings. Although these patients generally retain more autonomy, physiotherapy remains crucial for symptom relief, holistic support, and promoting continuity of care through active family engagement [21,22,23].

Understanding the specialized context of PC units, particularly in Portugal, highlights physiotherapy’s potential to support multiple stages of PC through personalized interventions [3,7,24]. Various therapeutic techniques have proven effective in promoting comfort: manual massage reduces anxiety, pain, and medication use. Therapeutic exercise helps manage fatigue, mood, pain, independence, and caregiver burden. Music therapy, hydrotherapy, and exercises with therapy balls aid relaxation and stress reduction. Integrative approaches, like acupuncture, aromatherapy, and mindfulness, alleviate pain, anxiety, and depression. Supportive technologies such as health apps and virtual reality also enhance well-being [24,25,26,27,28,29,30,31,32]. Professionals focus on relieving distressing symptoms, preserving autonomy, and providing compassionate and individualized care. In respiratory care, the aim is to ease breathlessness and enhance comfort through gentle bronchial hygiene and light exercises, involving caregivers and interdisciplinary teams to ensure comprehensive support and care [26,33].

However, physiotherapists and occupational therapists are still not formally integrated into the strategic framework of PC teams in Portugal. Integration remains inconsistent, and their roles are often undervalued [25,26,34,35]. Inclusion depends on team availability, contrasting with other countries in the European region where integration is established [11]. Key challenges include lack of role recognition, delayed referrals, absence of standardized protocols, and variability in methods and assessments. There is also a pressing need for greater investment in education, research, and policies grounded in evidence-based practice [26,31,36,37,38,39]. Additionally, interventions vary widely in structure, technique, frequency, and duration. Understanding this variability is essential to advancing the conceptualization and delivery of care [26,27,28].

In this scenario, there is still a long way to go before patients, both internationally and in the Portuguese context, can consistently benefit from comfort-focused interventions at the right time and place [3,11,13,21,22,23,40]. This study aims to contribute to a deeper understanding of how to strengthen the integration and clinical practice of physiotherapists within PC units, ultimately improving patient access to comfort-focused care throughout the illness trajectory.

Therefore, we aim to contribute to better understanding of the physiotherapist’s role in promoting comfort within the Portuguese PC context, from professionals’ perspectives, by (a) developing insights into the role of physiotherapy across PC units and (b) exploring perceptions and experiences related to their role in promoting comfort. This may serve as an asset in the national context and offer insights for international contexts, given the global variability in how PC is delivered.

## 2. Materials and Methods

### 2.1. Study Design

This study employed a descriptive qualitative approach, using online focus groups (FGs) designed according to the methodological framework proposed by Krueger and Casey [41]. To ensure methodological rigor, the study followed the Consolidated Criteria for Reporting Qualitative research (COREQ) checklist (see Supplementary Table S1) [42].

### 2.2. Study Setting, Participants, and Recruitment

The population consisted of all physiotherapists working in PC in mainland Portugal and the islands. A non-probabilistic convenience sample was used, with up to 10 participants planned for each of the three FGs, a number considered adequate for qualitative research [41,43], ideally totaling 30 participants for the study.

From this population, 21 participants were initially assigned to the FGs (seven per FG), based on availability, willingness to participate, and fulfillment of the following inclusion criteria. These inclusion criteria were internet access; a minimum of two years of professional experience; and at least one year of experience providing PC in one or more of the following settings, these being (1) in-hospital, (2) inpatient, or (3) community-based units. Eligibility was assessed by the lead researcher (V.V.P.).

Participants who did not meet the inclusion criteria were excluded [21,22,23]. Some who had previously confirmed their participation did not attend due to personal and professional reasons, most notably in FG 3, which was conducted with the minimum number of participants required (four) [41,43].

In total, 15 physiotherapists participated: five in FG1, six in FG2, and four in FG3.

### 2.3. Data Collection

The online FG sessions took place over a two-month period (December 2024 and January 2025) using the Zoom videoconferencing platform. Each session lasted approximately 60 min. Prior to each FG, participants completed a brief questionnaire to collect basic sociodemographic information. During data collection, all participants had their video and audio enabled, an arrangement negotiated and agreed upon in advance of each session. The sessions were recorded with participants’ informed consent and later transcribed to ensure an accurate analysis of the discussions and responses. Each session began with the presentation of a vignette on the concept of comfort, using the following definition:

Comfort can be defined as a condition experienced by individuals who receive care aimed at promoting it. It is characterized as an immediate and holistic experience of need satisfaction—whether active, passive, or cooperative—across three types of comfort: relief, ease, and transcendence, within four dimensions of human experience: physical, psycho-spiritual, environmental, and social [14].

Data were collected using a semi-structured interview guide with open-ended questions, allowing for a deeper understanding of participants’ perspectives and clearer insights into their perceptions and experiences (Appendix A). The guide was developed based on existing literature and the authors’ practical experience with the research topic.

The moderator (D.F.S.D.) and assistant moderator (V.V.P.) conducted the three FGs in a virtual meeting room appropriate for the study. The moderator ensured that relevant topics were addressed and encouraged all participants to contribute. Prompts used to elicit deeper responses included “Could you provide an example?”, “Please elaborate on your meaning”, and “Elaborate further”. Identical questions were used across all FGs.

Participants were actively engaged, often completing or expanding upon each other’s ideas, even when initial statements were incomplete. The atmosphere was positive, with moments of laughter and a sense of ease among participants.

Following the guidance of Krueger and Casey [41], the assistant moderator documented field notes during the interviews to thoroughly capture themes, major points, and pertinent comments. Furthermore, the field notes were used to record non-verbal behavior and to distinguish between speakers and their tone within the group. Towards the conclusion of the discussion, the assistant moderator delivered a concise overview of the salient points and prompted participants to add further remarks by posing a final open-ended question: “Is there anything else we should include?” This question is significant, as it can elicit other crucial discussion points [41]. All FGs were audio-recorded and transcribed verbatim by the main author immediately following each session. All FG transcriptions, in European Portuguese, were translated and then back-translated to ensure the original meaning was preserved.

### 2.4. Ethical Aspects

The study was approved by the Ethics Committee of the Polytechnic Institute of Leiria (CE/IPLEIRIA/92/2024, approved on 7 October 2024). Prior to data collection, all potential participants were apprised of the study’s aims and the option to withdraw from the study without repercussions, both orally and in writing. Consenting participants signed informed consent forms. No financial incentives were linked with participation. The moderator clearly explained the study’s purpose and the participants’ rights to confidentiality and anonymity. The study followed the ethical principles of the Declaration of Helsinki. The Zoom videoconferencing platform ensured the encryption of all FG sessions.

### 2.5. Data Analysis

The content of the responses was analyzed using Braun and Clarke’s reflexive thematic analysis, in alignment with the objective of the present study [44]. This approach was justified by its inductive, bottom-up method, which allowed for the identification and interpretation of themes emerging directly from the data. It also facilitated the recognition of both similarities and differences across responses, as well as the systematization of unexpected perceptions [44].

All FG interviews were imported and transcribed using the qualitative analysis software MAXQDA [version 24 (Release 24.8.0), VERBI Software, 2024, Berlin, Germany] to support data management.

According to Braun and Clarke [44], thematic analysis consists of six phases, which we followed carefully. The first phase involves familiarization with the data through thorough reading of all transcripts. The second phase consists of generating initial codes. Reading the transcripts allowed relevant information to be grouped into specific codes. In the third phase, these codes were organized into themes related to comfort in the context of physiotherapy interventions. The fourth phase involved reviewing the themes, including checking all codes against the full dataset. In the fifth phase, the themes were defined and named, resulting in the development of the final thematic map. Finally, in the sixth phase, thematic revision was used to guide the writing of the report.

Data coding was carried out by two co-investigators. The lead researcher (D.F.S.D.) coded all transcripts, which were then independently coded by two co-investigators with academic training and expertise in data analysis (V.V.P. and A.Q.). In cases of coding discrepancies, consensus was reached through discussion. Regular cross-checking among the co-authors ensured that the codes did not reflect the interpretation of a single individual. All authors participated in the verification and refinement of the themes and domains.

The participants’ personal data were anonymized in the material, and access was restricted solely to the writers. All obtained data were stored in a digital folder with access restricted to the lead investigator. Archives will be destroyed one year after the study’s conclusion. To safeguard privacy and consensus among participants, alphanumeric codes were associated with data quotes. Excerpts from the participants’ discourse were numbered according to the FG in which they took part, considering the type of team (In-Hospital—IH; Inpatient—I; or Community—C), sex (XX/XY), and the participant’s assigned number within the group [44].

### 2.6. Trustworthiness

The research team was composed of three investigators: the principal investigator (Master’s student) and two co-investigators (Ph.D.), both of whom oversaw the FG interviews and had prior experience in conducting FGs and qualitative research. One of the co-investigators served as the session secretary during the FGs.

The student was informed by the research team about the objectives and purpose of the study, collaborating and making decisions jointly throughout the process.

The interviews were fully transcribed and identified. The research team engaged in ongoing discussion regarding data collection, saturation, and the emerging findings throughout the process. The principal investigator (D.F.S.D.) is a female physiotherapist working in PC and continued care, with practical expertise in supporting individuals with PC needs. The other researchers have competence in conducting research in the field of PC, as well as proficiency in qualitative inquiries (A.Q. and V.V.P.). V.V.P. is an occupational therapist and associate professor with expertise and practice in rehabilitation settings. These varied experiences enriched the understanding of the material and mitigated any potential biases.

## 3. Results

### 3.1. Sample Description

Of the 15 participants, 12 were women, with ages ranging from 25 to 60 years, the majority falling between 31 and 50 years old. The participants were based in six regions of the country (Azores, Guarda, Lisbon, Porto, and Santarém), with most working in the Lisbon district and near the Portuguese coast. Only Guarda represented a region from the interior of Portugal.

All participants had at least one year of experience providing PC, with most reporting between one and five years of experience. A total of 12 out of 15 had received advanced training in PC. The findings reveal that physiotherapists are not formally embedded in PC teams. Most reported contributing up to 10 h per week to PC teams, approximately one-third of a standard 35 h workweek. A few reported more than 10 h, and only one participant worked over 21 h weekly. This collaboration fluctuates depending on referral volume and is provided on an as-needed basis.

The study included physiotherapists working within both the public and private sectors of the healthcare system. This included, for example, professionals collaborating with community-based PC support teams. Regarding the professional composition of the PC teams in which the physiotherapists worked, most teams were composed of medical and nursing staff. These were followed by professionals in social work, psychology, dietetics and nutrition, occupational therapy, and speech therapy. In community settings, teams also included music therapists and complementary medicine practitioners. In all unit types, the presence of a spiritual care provider was reported.

### 3.2. Themes and Subthemes

The data were organized into seven themes, divided into subthemes. For each theme, we described the central aspect (Table 1).

#### 3.2.1. Challenges

Upon data analysis, seven themes were identified in relation to the contribution of physiotherapy to comfort in PC. The first theme concerns the ‘Challenges’ faced by physiotherapists.

In in-hospital care units, personal barriers are particularly prominent. These include, above all, the lack of awareness among many healthcare professionals and teams regarding the role of physiotherapy in PC, often leading to its underutilization. Despite the broad potential of physiotherapy in promoting comfort within PC, the success of physiotherapists’ involvement depends largely on coordinated efforts with both the care team and the patient’s family, while always adjusting to the clinical condition.

“*The challenge is integrating physiotherapy into the hospital care plan. Many still see our role solely in terms of rehabilitation, not in symptom relief or comfort.*”

(FGIH-XY-4).

“*One barrier is that professionals are unaware of the benefits physiotherapy can offer both patients and families. We need to build knowledge.*”

(FGIH-XY-5).

Another personal barrier is cultural resistance, which contributes to the lack of understanding about PC. It is necessary to demystify what it means to be a palliative patient and the possible scope of physiotherapy within this context. Among teams, and even physiotherapists themselves, there persists a belief that little can be done in PC.

“*We, as physiotherapists, need to demystify the idea of who a palliative patient is. We need to shift from the mindset of ‘there’s nothing to be done’ or that a simple mobilization is enough, I must actively seek knowledge. The notion that ‘there’s nothing to be done’ is wrong, but it still exists among teams and physiotherapists.*”

(FGHI-XX-7).

Further financial and political barriers include the absence of government guidelines supporting the inclusion of physiotherapy in PC, and the lack of public policy formally authorizing the full integration of physiotherapists within interdisciplinary PC teams.

“*We face the barrier (…) of not being integrated into the team in the same way as other professional groups.*”

(FGIH-XY-5).

As a result, the aforementioned obstacles give rise to organizational barriers. Physiotherapy is still not considered an essential component of PC, which hinders both funding and patient access, mainly due to the lack of integration into the healthcare system. For example, patients typically gain access to physiotherapy in PC only after a physician’s referral, a standardized procedure that, when combined with limited understanding of the timely benefits of physiotherapy, can delay or even prevent referrals.

“*We can only begin an intervention if there’s a physician’s request. (…) That’s why having the physiotherapist integrated into the inpatient care team would be more beneficial for the patient. When physiotherapy is delivered sporadically, there is no opportunity for proper monitoring and follow-up.*”

(FGIH-XY-4).

Barriers within the health system were also identified, particularly those related to inadequate training. Most physiotherapy degree programs do not include coursework on PC, which limits professional preparedness. Even at an advanced level, the number of trained professionals remains low, resulting in a shortage of physiotherapists with specialized expertise.

“*We must work towards becoming part of the PC team, but adequate training in undergraduate and advanced education makes all the difference.*”

(FGHI-XX-7).

In hospital-based PC units, physiotherapy is generally provided only after a direct request from medical or nursing staff. As a result, physiotherapists are often called upon to intervene in advanced stages of illness, and they are not formally integrated into the team:

“*The teams are made up of physicians and nurses. Other professionals are called in to intervene with the patient based on specific needs. The level of knowledge about what physiotherapists can do may influence whether they are referred or not.*”

(FGIH-XY-4; FGIH-XX-7).

In inpatient PC units, personal barriers also stem from lack of knowledge. Many healthcare professionals remain unaware of the potential contribution of physiotherapy in this setting, resulting in underutilization. This lack of PC literacy constrains the physiotherapist’s role, as other team members fail to recognize its benefits in symptom management and, by extension, patient comfort. Another personal barrier involves cultural resistance, specifically the widespread misconception that PC is limited to end-of-life care, often resulting in delayed referrals for physiotherapy. The lack of palliative literacy among health teams significantly impacts timely patient identification and referral.

“*(…) The biggest issue is the lack of palliative literacy among healthcare professionals. PC is not just end-of-life care, they have no idea how it works or what the benefits are (…) There’s a major problem with clinical community literacy.*”

(FGI-XX-6).

Organizational barriers were also cited, particularly those related to the insufficient integration of physiotherapy into the healthcare system. Since physiotherapy is not yet regarded as an essential component of PC, this limits both funding and accessibility. In line with previous observations, patients are often referred late in the disease trajectory, at a point of severe symptom instability, when they no longer meet the minimum conditions for physiotherapy, either because they are too debilitated or because it is simply no longer the appropriate time.

“*(…) Unfortunately, many patients are still referred too late and arrive at the unit in their final days (…) While intervention is possible at end of life, ideally it should begin earlier. When patients arrive in such advanced stages, with uncontrolled pain, it’s hard to even approach them due to the level of distress.*”

(FGI-XX-6)

In long-term inpatient settings, physiotherapists tend to work with greater autonomy. Although collaboration with the team may be more accessible, referrals still dictate involvement, most often at the end-of-life stage. They are not formally integrated into the team:

“*Patients are referred by the team after an initial screening (…) when we are called in, it’s often for patients in their final hours (…) they arrive at the unit and end up passing away, leaving us with the feeling that much more could have been achieved.*”

(FGI-XX-3).

In community-based PC units, personal challenges were also reported, primarily a lack of knowledge, cultural resistance, and undervaluation of physiotherapy. Both teams and patients/caregivers often do not fully understand the importance of functional rehabilitation, which compromises referral and adherence, making expectation management among all stakeholders particularly difficult. Participants also noted the challenge of aligning the expectations of families and caregivers, who may hold unrealistic beliefs about the outcomes of physiotherapy, necessitating a dual focus on both the patient and their support network. At the same time, the lack of recognition and proper integration within PC teams can result in a sense of isolation, which hinders effective collaboration.

“*(…) Although I’m not formally included in a PC team, I believe we should be in communication with them so we know what the common goal is, and that’s a challenge for us, and it doesn’t always happen.*”

(FGC-XX-2).

Regarding cultural resistance and the misconception that PC is limited to the final stage of life, this can lead to delayed physiotherapy initiation and complicate the conditions under which physiotherapy is delivered, making it more difficult to adhere to clinical procedures.

“*(…) Of course, in this context, we have to assess the patient’s needs on a daily basis, understand their clinical status, and adapt the intervention accordingly. Exercise is what I use in a more standardized way, adjusted to the patient’s condition, but often the patient doesn’t recognize its importance. Working with the family becomes challenging (…) especially in managing family expectations. (…) Educating the family is essential, as it allows us to share our strategies with those who will be there, empowering them to support the patient’s needs.*”

(FG-XX-3; FGC-XX-4).

“*The family should be both active participants in care and recipients of it.*”

(FGC-XX-2).

Other barriers encountered in the community setting are organizational in nature, particularly the lack of integration into the healthcare system due to physiotherapy not being recognized as a core component of PC, thereby limiting funding and accessibility. Additional systemic barriers include inadequate training and the low number of specialized physiotherapists in community settings. Proper education contributes significantly to the effectiveness of PC interventions, especially in addressing more complex aspects such as existential suffering, hopelessness, and spiritual issues, all of which require sensitivity and advanced communication skills to foster a sense of comfort.

“*(…) Spirituality (…) can provide great comfort to patients when they see that the person in front of them isn’t afraid to talk about the meaning of life, of death, of vulnerability, and we can show that we are open to that. It can be deeply comforting, and it’s much easier when done as a team.*”

(FGC-XX-2).

In community-based settings, physiotherapists work even more independently, not always dependent on referrals (they may be initiated by family). Contact with the core PC team occurs primarily when clarification is needed or when it is necessary to align treatment goals, but this professional is not formally integrated into the team:

“*Although I’m not formally integrated into a PC team, I believe we should be in closer communication with the team to understand the shared goals. That would make things less challenging for us.*”

(FGC-XX-2).

#### 3.2.2. Diseases

The second theme is related to ‘Diseases’ treated by physiotherapists. All physiotherapists from the three FGs reported providing care to both oncological and non-oncological conditions. Overall, there was a predominance of non-oncological cases compared to oncological ones.

Within in-hospital units, however, physiotherapists reported a greater number of oncological cases, often in advanced or terminal stages, as well as frailty and elderly patients:

“*I can speak from my professional experience in a hospital context. More than half of the patients are oncology cases (…) then neurological conditions, heart failure, cases of organ failure, and COPD.*”

(FGIH-XY-6).

“*The overwhelming majority of cases involved terminal cancer, as well as some neurological conditions, but most, if not all, were advanced, progressive, and incurable diseases in their terminal phase.*”

(FGIH-XY-4).

In the inpatient units, there appears to be a balance in the care provided to both oncological and non-oncological conditions, with a strong focus on high frailty:

“*Oncological cases, but also numerous ALS, strokes, and dementias.*”

(FGI-XX-7)

“*A significant focus is on oncology, head, neck, brain (glioblastomas), lung, and breast cancers (…), as well as many cases of dementia (…) and situations of severe frailty. Then there are chronic conditions, (…) COPD (…) ALS and multiple sclerosis (…) extensive hemorrhagic strokes. These are individuals in a state of vulnerability, which may not be the official reason for admission, but is often the underlying cause.*”

(FGI-XY-4).

In the community PC units, there appears to be a stronger focus on non-oncological diseases, especially neurological conditions:

“*In my practice, I mainly work with respiratory conditions such as COPD, respiratory failure, neurodegenerative diseases, and some terminal oncological cases (lung and breast cancer).*”

(FGC-XX-2).

“*In my case, I mostly deal with dementia, neurodegenerative diseases, and terminal oncological conditions such as breast and lung cancer (…) COPD, as well as cardiac and respiratory failure.*”

(FGC-XX-5).

In this setting, in addition to what has already been described, it is important to highlight that most patients are elderly or very elderly, often presenting with significant frailty and marked functional decline.

“*Patients present with respiratory and palliative conditions and are very elderly.*”

(FGC-XX-2).

“*Dementia and frailty are common among the older population, particularly the very elderly.*”

(FGC-XX-3).

#### 3.2.3. Signs and Symptoms

The third theme refers to the ‘Signs and symptoms’ in which physiotherapy appears to have an impact in terms of comfort-focused interventions within PC.

In the inpatient and hospital-based FGs, the most frequently mentioned signs and symptoms included pain (acute and chronic), fatigue, dyspnea, immobility syndrome, nausea, vomiting, and edema. As one participant explained,

“*Pain, dyspnea, fatigue/asthenia (…) decreased mobility as both a sign and a symptom—these are the main reasons why the medical team refers patients to physiotherapy.*”

(FGIH-XY-6).

Within the hospital-based inpatient care setting, the primary symptoms mentioned were mostly respiratory in nature, such as cough and secretions, as well as digestive symptoms, particularly constipation:

“*I mostly work with oncological cases, but increasingly with organ failure—cardiac, pulmonary, hepatic, as well as neurological conditions and ALS. These situations lead to both respiratory and digestive difficulties.*”

(FGIH-XY-6).

Additional cumulative signs and symptoms emerged in the inpatient setting, such as muscle rigidity and spasticity, loss of balance and immobility, and risk of complications such as pressure ulcers.

“*Lack of strength, reduced functional mobility, and balance impairment.*”

(FGI-XY-2).

Psychological signs and symptoms such as depression, emotional distress, anxiety, and fear were consistently highlighted in the community-based FG. Dyspnea, pain, intense fatigue, and muscle weakness were also reported:

“*I’d like to add depression (…) it limits movement, and our role is to work towards achievable goals that restore hope, motivation, and comfort.*”

(FGC-XX-5).

“*I’m not sure if this fits under the category of symptom (…) but what people really feel is hopelessness, experienced in a suffering body (…) it’s truly intense, leading to depression and discouragement. We need to address this by setting short-term goals that help the body feel more pleasurable to inhabit.*”

(FGC-XX-3).

Other signs and symptoms mentioned across all FGs included constipation, lymphedema and edema, motor coordination difficulties, urinary incontinence, sarcopenia, hemiparesis, and organ failure.

“*Lower limb edema can cause significant discomfort (…) we can intervene with massage (…) and help maintain balance.*”

(FGI-XX-8).

Regarding motor coordination, one participant noted:

“*(…) Patients feel distressed when they lose proper motor coordination (…) due to the resulting difficulties in daily living activities and changes in gait pattern. It leads to urinary incontinence, edema, and immobility.*”

(FGIH-XY-4).

#### 3.2.4. Family and Caregivers

The fourth theme refers to ‘Family and caregivers’, concerning the role of family members and caregivers, who are essential to the physiotherapist’s work in providing comfort in PC. Still, it was understood that their involvement is highly dependent on the setting in which care takes place, resulting in family and caregivers being more or less active participants in the process.

In in-hospital care units, all professionals confirmed that interaction with family members rarely occurs during patient encounters; interaction and collaboration are crucial but remain limited.

“*I used to love doing sessions with the relatives present, I really did, even if just to get the patient to engage more or to carry out a more meaningful activity for them. Having the relatives there can make the sessions more interactive (…) the presence of family is a facilitator, not a barrier.*”

(FGIH-XX-3).

In inpatient units, family member and caregiver involvement does occur, but its form and timing vary significantly. It appears that integrating family members into physiotherapy care still falls short of what is considered essential, with significant variations across units.

“*(…) We need to provide training to the family, some relatives want to be fully involved in the care. I wish we had more family meetings with the team in my unit to better align expectations.*”

(FGI-XX-7).

In the community-based units, by nature, family members and caregivers are almost always present at the site of care. However, this does not necessarily mean that their participation is effective, as numerous challenges arise in a setting that tends to favor the patient but less so the healthcare professional. On this topic, one participant shared that

“*It’s not easy, but it’s a beautiful process (…) the family should be active in care, but also recipients of care themselves.*”

(FGC-XX-2).

#### 3.2.5. Techniques

The fifth theme refers to ‘Techniques’ employed by physiotherapists.

In in-hospital care units, professionals reported the use of therapeutic massage (for relaxation) and thermotherapy (application of heat or cold) to manage pain, enhance patient comfort, and alleviate muscular and joint pain. They also emphasized the importance of patient positioning, particularly in bed and in wheelchairs, as a strategy to prevent pressure ulcers. Other commonly used approaches include passive and active-assisted mobilization, as well as passive and active-assisted stretching, aimed at preventing stiffness and muscular shortening. Participants also mentioned adapted therapeutic exercise to help maintain mobility and prevent secondary complications. This included basic functional movements to support residual autonomy, light joint mobility exercises to preserve function without inducing fatigue, and balance and coordination training to reduce fall risk.

Sensory stimulation, such as gentle touch, aromatherapy, and music therapy, was reported to complement mobilization and movement.

“*It can be as simple as playing a favorite song to provide comfort, or adjusting the environment so the person can watch their favorite show comfortably, read a book, or sit by the window (…) simple things that are deeply meaningful to the patient, even if not strictly physiotherapy techniques.*”

(FGIH-XX-7).

“*Positioning, relaxation techniques and music, therapeutic exercise, mobilization, lymphatic drainage (…) energy conservation techniques.*”

(GFIH-XX-3)

“*I use therapeutic exercise with the patient, and I can also teach it to the caregivers. It’s helpful for physical reconditioning, encouraging the patient to engage in meaningful activities and maintain autonomy in daily tasks.*”

(FGIH-XX-4).

“*Training for a specific activity, aligned with the person’s goals, what’s meaningful to them, activities involving movement that make sense.*”

(FGIH-XY-5).

To address dyspnea and enhance pulmonary function, participants use respiratory rehabilitation, including diaphragmatic breathing training and breath control techniques to reduce respiratory effort. These are often combined with assisted coughing, postural drainage for secretion clearance, incentive spirometry to improve oxygenation, and non-invasive ventilation.

“*All of these techniques aim to relieve dyspnea. Non-invasive ventilation strategies aren’t meant to correct respiratory failure, but to reduce the work of breathing (…) airway clearance techniques are meant to provide comfort. Positioning is used to optimize ventilation and reduce dyspnea, contributing significantly to the patient’s comfort.*”

(FGIH-XY-5).

In addition, participants emphasized the importance of motivational and psycho-emotional support, particularly in helping patients accept their health condition and set realistic goals. Interdisciplinary teamwork also plays a key role in supporting both emotional well-being and effective communication with patients and families.

“*Communication is crucial, especially with specific training in palliative care. The way we say things—or don’t say them—and how we use verbal and non-verbal communication has a huge impact. Communication can be an excellent tool, or it can become a weapon with a negative impact.*”

(FG-XY-6).

In inpatient units, adapted therapeutic exercise was the most frequently mentioned method (aimed at maintaining or improving mobility), along with relaxation techniques to relieve pain, muscle tension, constipation, anxiety, and dyspnea, particularly when driven by anxiety and fear.

“*We can have a significant positive impact on patients experiencing anxiety and uncontrolled dyspnea through relaxation techniques.*”

(FGI-XX-6).

“*The real difference lies in what is meaningful to the person, being able to stand up to reach for something, or simply a movement while eating. A specific position or a massage, even something as basic as a touch (…) or strength training to relieve pain.*”

(FGI-XX-8; FGI-XX-3).

Positioning in bed or wheelchairs is intended to relieve pain and prevent pressure ulcers, while passive and assisted mobilization helps prevent muscle shortening and joint stiffness and improve constipation.

“*Simple but highly effective, positioning makes a huge difference. Our physiotherapy perspective really matters (…). If discomfort is caused by constipation, sometimes just a bit of mobilization, a few active exercises, or gait training can bring immediate relief.*”

(FGI-XX-6; FGI-XX-4).

Electrotherapy techniques such as TENS and pressotherapy, combined with lymphatic drainage and compression bandaging, were also mentioned as effective tools for pain and edema relief. Thermotherapy was again noted for its role in reducing muscle tension and pain.

“*I believe every technique available to us can be applied in palliative care, depending on the patient’s goals and to alleviate symptoms—TENS, pressotherapy, bandaging, drainage… whatever we can use. Even strength training can help relieve pain.*”

(FGI-XX-3).

Respiratory rehabilitation was also used to manage dyspnea, particularly through airway clearance in cases of respiratory infections or aspiration pneumonia.

“*Respiratory symptoms go beyond dyspnea, they include bronchial obstructions and other causes (…) I deal mostly with anxiety-driven dyspnea, but respiratory infections and aspiration pneumonia are the most common situations I intervene in.*”

(FGI-XX-6; FGI-XX-7).

Participants also reported using motivational and emotional support strategies, especially active listening and fostering hope. Defining realistic goals and managing expectations in relation to the patient’s meaning of life, and that of the family, was considered crucial.

“*We have a distinct sensitivity when it comes to comfort, when there’s hopelessness or a lack of meaning, that’s where we step in (…) and we often do it through communication, which is our tool.*”

(FGI-XX-5).

“*Listening is what matters. I’ve had interventions where I never physically touched the patient but spent an entire hour just listening to what they needed to share in that moment (…) and that, to me, is a valid and meaningful intervention.*”

(FGI-XX-4).

In community care units, participants most often mentioned therapeutic massage and compassionate touch, used for relaxation, relief of chronic pain, and reduction in muscle tension, all crucial for managing psychomotor agitation and supporting emotional well-being.

“*Massage has had transcendent effects. I try to create the most relaxing environment possible, and the touch is compassionate—it’s about presence and intention.*”

(FGC-XX-3).

For dyspnea control, specific techniques are used to ease the sensation of breathlessness, with ventilatory support used when necessary. Participants also apply targeted breathing techniques to improve ventilation and comfort.

“*For obvious reasons, dyspnea control. When someone is breathless, there is no comfort.*”

(FGC-XX-2).

Therapeutic exercise and mobility were especially relevant in the patients’ daily lives. Participants used adapted exercises for muscle strengthening, function maintenance, stretching, and exercises to combat fatigue and sarcopenia.

“*The goal remains to provide comfort to the patient, even if it’s just a change in position. Movement brings comfort (…) just the fact that mobility exists.*”

(FGC-XX-2; FGC-XX-3).

Music and dance were also widely used. Assisted and spontaneous dancing served as powerful stimulants of movement, promoting relaxation, emotional regulation, and enhanced comfort.

“*I use more organic movements, often incorporating music and dance, dance as an expression of natural movement (…) the person may start with assisted dancing, then they begin to lead the movement themselves. This can be done lying down or standing. I use a lot of relaxing music (…) relaxation really helps with fatigue and pain.*”

(FGC-XX-3).

Regarding support for caregivers and family, participants unanimously emphasized the importance of training in patient mobilization, involving family in care to reduce burden, therapeutic massage, and direct psycho-emotional support for caregivers. In terms of emotional and spiritual support, active listening and therapeutic presence were essential to address emotional concerns and open dialog around spirituality, helping to restore a sense of meaning and comfort. Manual lymphatic drainage was reported to help manage fatigue and pain, while techniques for constipation and nausea were also widely used.

“*When applied with precision and sensitivity, manual lymphatic drainage not only reduces edema and improves circulation, but also provides comfort and well-being, it’s an essential technique in palliative care.*”

(FGC-XX-2).

“*Families should be integrated into care, as active participants in caregiving, but also as recipients of support, because caregivers often need care themselves.*”

(FGC-XX-4).

#### 3.2.6. Equipment and Materials

The sixth theme focuses on the ‘Equipment and materials’ used in physiotherapy within PC to address comfort.

In the in-hospital setting, participants highlighted the need to adapt materials to the reality of PC, prioritizing both patient comfort and functionality while carefully avoiding excessive fatigue. Among the most referenced resources were the pedal exerciser, which helps promote circulation and prevent joint stiffness in the lower limbs with minimal effort; walking aids such as canes, walkers, and wheelchairs, which enhance stability and safety during mobility; and orthoses, used to prevent deformities, support postural alignment, and reduce pain.

Less frequently mentioned was the use of cushions and postural supports (aimed at preventing pressure ulcers and ensuring patient comfort); therapy balls, elastic bands, sticks, dumbbells (for light mobility and muscle or joint strengthening exercises); and respiratory rehabilitation devices (such as incentive spirometers and materials used in postural drainage techniques). Regarding this topic, participants reported the following:

“*In terms of equipment, there wasn’t much, only what I could bring with me to the hospital room: sticks, pedal exercisers, dumbbells, balls.*”

(FGIH-XY-7).

“*Spinal orthoses, in cases of bone tumors with bone stabilization, for example, are the most frequently used (…) when there is distal neurological dysfunction, they are also used occasionally (…) to maintain a specific position when there is associated pain.*”

(FGIH-XY-5; FGIH-XY-6).

“*(…) Devices we have to assist with positioning (…) positioning cushions, silicone pieces that contribute significantly to ventilation management (…) equipment for secretion removal.*”

(FGIH-XY-5).

“*We use walking aids and orthoses, these tools help provide some comfort and improve quality of life; it’s a multifactorial and individualized response.*”

(GFIH-XY-6).

In the inpatient units, the equipment and materials most frequently cited by participants included pedal exercisers, walking aids, sticks, balls and weights, and electrotherapy as an adjunct tool, including TENS, pressotherapy, and thermotherapy.

“*We rely on mechanical, electrical, and thermal devices such as TENS (…). We use weights, sticks, walking aids, creams, balls, positioning devices, oximeters, pedal exercisers (…) walking aids, ankle weights, free weights, sticks, balls, and hands.*”

(FGI-XX-3; FGI-XX-7; FGI-XX-8).

Special emphasis was also placed on the use of positioning cushions and respiratory rehabilitation devices. Positioning cushions are employed to optimize patient posture, enhancing pain relief, preventing pressure ulcers, and improving ventilation. Respiratory rehabilitation devices are used for monitoring and optimizing interventions aimed at reducing dyspnea and maintaining bronchial hygiene; examples include oximeters and stethoscopes.

“*If I had to name the materials I use the most… probably those related to respiratory care, like oximeters and stethoscopes. I also love working with pedal exercisers, and patients usually respond well. I often use resistance tools like weights or elastic bands, though normally I just adapt to the surrounding environment.*”

(FGI-XX-6).

In community-based care units, participants reported the use of the following equipment and materials: speakers and music, therapy balls and elastic bands, compression therapy devices, non-invasive ventilation equipment, cough-assist and bronchial clearance devices, treadmills, electrotherapy units (TENS and pressotherapy devices), and the piano.

Therapy balls and elastic bands are used for customized, adaptive exercises focused on muscle strengthening and mobility. Compression devices are used for lymphedema management. Non-invasive ventilation and devices for cough assistance and bronchial clearance are highlighted as essential for respiratory support. Treadmills are used selectively, depending on the patient. Electrotherapy (TENS and pressotherapy) is applied occasionally. Music and sound systems are used both for relaxation and to encourage movement, with the type of music adjusted to individual needs. Finally, the piano is employed as a therapeutic tool to stimulate movement.

“*In my practice, I rely heavily on my hands (manual therapy). I use TENS, balls, weights, bands, heat and cold therapies, and compression devices.*”

(FGC-XX-4).

“*In respiratory care, I use devices for cough assistance, bronchial clearance, ventilatory support, and some strength training tools.*”

(FGC-XX-2).

“*What I use the most is music, my speaker is my real working instrument, in addition to my hands.*”

(FGC-XX-3).

#### 3.2.7. Format

The seventh theme relates to ‘Format’.

In in-hospital care units, all participants reported that both the format and dosage of physiotherapy interventions are flexible and individualized, considering the patient’s clinical condition, fatigue, and tolerance. There is no rigid protocol; instead, interventions are adjusted according to patient tolerance and goals, aiming to minimize symptoms without causing undue exertion. Sessions are delivered on an individual basis, tailored to the patient’s functional capacity and often utilizing assistive equipment to ensure comfort and manage fatigue. Continuous supervision plays a key role in determining both format and dosage and is generally provided by the available healthcare team, including physicians and nurses.

In terms of session duration, interventions are brief and adaptable, ranging from 15 to 60 min, depending on the patient’s fatigue level and response. Frequency typically does not exceed once or twice per week. As for location, physiotherapy is most often conducted at the patient’s bedside in the ward, although it may occasionally take place in the hospital’s physical medicine and rehabilitation gym facilities.

“*I work on the wards, and I have to take care to ensure personal protection and privacy, cover the patient with a sheet, understand how the patient is feeling, whether they’re comfortable or not (…) we have to find the right balance to achieve gains.*”

(FGIH-XY-4).

Regarding dosage, it is determined by the patient’s response. Both intensity and quantity are adjusted according to symptomatology, with the goal of avoiding excessive strain and fatigue. Intensity is based on gentle, progressive movements that respect the patient’s pain and discomfort threshold.

“*I think daily assessment is essential in these patients, and our intervention depends on that. (…) So, we have to strike that balance to achieve gains, maintain the patient’s quality of life, and keep them motivated.*”

(FGIH-XY-7).

“*(…) The most important thing is symptom management (…) ensuring the patient’s comfort. If the intervention doesn’t align with that, we may need to reassess and quickly decide whether to stop or try something else.*”

(FGIH-XY-5).

“*I prefer to ensure personal protection and privacy, (…) it´s almost impossible to be worried with the dosage*”

(FGIH-XY-4)

Individual sessions are focused on comfort and tailored to the patient and are held in wards or occasionally in rehabilitation gyms. Duration: 15 to 60 min, adjusted for fatigue. Frequency: 1–2 times per week. Dosage: Gentle movements guided by symptom burden.

In the inpatient context, the format remains individualized. The session duration is short and flexible, typically ranging from 15 to 60 min, and is adapted according to the patient’s fatigue and responsiveness. Frequency is limited to once, or at most twice per week. While interventions are usually performed in patient rooms, the institution’s gym or designated therapy spaces may also be used.

“*There are no standard formulas. We ensure comfort and symptom control through a plan that reflects what is meaningful to the patient in that moment. (…) One day the patient may be doing great, the next day terribly. One day they manage an excellent walking session and then spend a week unable to move. (…) It’s about applying the evidence from other areas and adapting it to the specific reality of each patient, being sensible.*”

(FGI-XX-6).

As for dosage, the number of sessions is guided by what the patient needs to achieve, presented as mutually agreed-upon goals.

“*The frequency, the amount, I believe it’s a balance between what is meaningful for the person and how many sessions are needed to achieve what they need, or the time involved. It’s this link between two things: what they need and what matters to them. (…) A balance between these two, an exercise that requires a certain frequency to develop a specific skill.*”

(FGI-XX-8).

Individual sessions are performed in patient rooms or the gym. Duration varies from 15 to 60 min, adjusted for fatigue and response. Frequency: 1–2 times per week. Dosage is balanced according to meaningful therapeutic goals.

In the community setting, it was noted that comfort-focused physiotherapy does not follow a rigid protocol and is adapted to the patient’s condition and specific needs. The only intervention reported to allow for a structured approach to format and dosage was therapeutic exercise, which is tailored to individual needs, avoiding fatigue and prioritizing comfort. Sessions in this context are generally longer, lasting up to ninety minutes, as they include time for travel and often involve the family, whose presence requires additional attention and interaction.

“*The only non-pharmacological physiotherapy intervention I use that is dose-dependent is therapeutic exercise, in terms of both dosage and frequency according to patient needs.*”

(FGC-XX-5).

This exercise is individual, flexible, and based on the patient’s condition, with no rigid protocol: performed at home. Therapeutic exercise is the only approach suitable for dosage. Duration: up to 1.5 h, accounting for travel and family involvement.

## 4. Discussion

The results of this study highlight the fundamental role of physiotherapy in promoting comfort within PC across various care settings: inpatient hospital units, inpatient units, and community-based units. This study addresses a gap by exploring physiotherapy’s role in Portuguese PC through qualitative FGs with 15 physiotherapists. It offers valuable insights into practice, challenges, and strategies for improved integration, but it is limited by its small, regionally confined sample and exclusive focus on physiotherapists. Future research should include broader geographic areas and additional stakeholders to better inform policy and clinical decision-making.

The data revealed key themes that identified the main challenges, methods, and strategies used in physiotherapy, while also confirming the diversity in how physiotherapists are integrated into PC teams within Portuguese healthcare context, as seen in other countries worldwide [3,7,8,9,10,11,12,13].

This study reveals physiotherapy as a vital yet underrecognized component of PC, with a growing and complex role that requires systemic support through education, policy, and interdisciplinary integration. Addressing current barriers and investing in holistic, patient- and family-centered approaches are essential steps to advance comfort-focused physiotherapy interventions within both national and international PC frameworks. Clearly, this field places strong emphasis on symptom management and the maintenance of functional capacity [2,3,4,5,6].

This study enabled the exploration of physiotherapeutic interventions in both oncological and non-oncological patients across different settings, including home care, a context in which little is known about the contribution of physiotherapy to comfort in PC. Identifying settings with varying prevalence of oncological and non-oncological conditions provided a clearer understanding that the specific needs of these populations differ. Consequently, the therapeutic interventions employed by physiotherapists are tailored not only to the pathology but also to the specific care setting [2,3,4,5,6].

The study highlights that physiotherapy in PC extends beyond traditional cancer-related contexts, encompassing significant involvement with non-oncological diseases such as advanced neurodegenerative and respiratory conditions. This diversification underscores the need for physiotherapists to tailor interventions to varying patient populations and care environments (hospital, inpatient, community), reflecting the evolving demands of PC. It presents an opportunity to rethink the scope of physiotherapy and develop competencies aligned with these broader roles, reinforcing the urgent need to integrate PC training at both undergraduate and advanced levels. Although participants had advanced training, there was a consistent call for more specific preparation to address complex issues such as existential suffering, emotional support, and spiritual care for both patients and families, particularly relevant in community settings [3,7]. This points to an urgent need for curriculum reform and continuous professional development to equip physiotherapists with the skills required for holistic care, especially in emotionally and spiritually demanding contexts such as community-based care [23,24,27].

Late integration of physiotherapists into PC teams, limited institutional support, and insufficient recognition of their role impede timely and preventive care. The study confirms that structural and educational barriers persist globally, restricting access for patients who require comfort-focused interventions. These barriers reflect systemic shortcomings that negatively impact care quality and patient outcomes, emphasizing the urgent need for policy reform and institutional guidelines to formalize physiotherapy’s role within PC. The weekly time each professional dedicates to PC is generally limited, mostly up to 10 h, reflecting the lack of institutional and strategic investment, underpinned by a still narrow understanding of physiotherapists’ role in this field [11,21,22,25,26,27,34,35].

Physiotherapy’s central contribution lies in managing symptoms such as pain, dyspnea, fatigue, and mobility impairment, crucial for patient comfort and quality of life. The use of diverse techniques—from respiratory rehabilitation to therapeutic exercise and psychosocial support—reflects an integrative, patient-centered approach aligned with the philosophy of PC. Recognition of emotional and psychological symptoms further highlights the need for holistic care models that address the full spectrum of patient and caregiver needs [3,12,13,26,28]. This field clearly places strong emphasis on symptom management and the maintenance of functional capacity, which are essential to support comfort-focused interventions.

To manage symptoms and promote comfort, physiotherapists use a range of techniques, including respiratory rehabilitation, pain and mobility therapies (e.g., massage, thermotherapy), and functional support through mobilization and therapeutic exercise. Assistive devices such as canes and wheelchairs are tailored to patient needs, with an emphasis on minimizing fatigue. Comfort is further enhanced by tools like pillows, music, dance, and therapeutic touch. While music was mentioned as a complementary method, other approaches such as acupuncture, aromatherapy, and digital technologies were not mentioned [24,25,26,27,28,29,30,31,32].

Physiotherapy in PC adapts both the format and dosage of interventions, which are mostly individual, last between 15 and 60 min, and occur up to twice weekly, with a focus on comfort and symptom relief. In community settings, therapeutic exercise sessions may extend up to 90 min, accounting for travel time and family involvement.

This study highlights family and caregivers as essential partners and recipients of care, especially in home-based PC. Their active involvement and the challenges they face necessitate targeted education, emotional support, and therapeutic guidance from physiotherapists. This emphasizes that effective PC requires addressing the caregiver–patient dyad, extending physiotherapy’s scope beyond direct patient interventions to include caregiver well-being [3,11,12,13,24].

Interdisciplinary teamwork is acknowledged as vital for coherent care delivery, yet the study reveals inconsistencies in its implementation, particularly in community settings where physiotherapists often work in relative isolation. Strengthening collaborative practices is critical to aligning therapeutic goals and improving patient outcomes, suggesting that institutional support for integrated models of care is needed [1,8,27]. Nevertheless, a stronger sense of team belonging and shared goals appears to be more consistent in the Azorean context [31,36,37,38,39].

Overall, the findings highlight the need to develop equitable health policies, promote specialized PC training, and strengthen interdisciplinary collaboration. These efforts are essential to optimize physiotherapy’s impact on comfort and quality of life in PC. Furthermore, expanding research to include broader geographic regions and the perspectives of other healthcare providers, patients, and caregivers will enrich understanding and inform effective care models. Such steps are critical to adapting and implementing sustainable approaches that ensure timely, comprehensive access to PC and its various domains, addressing the specific needs of patients and families [1,8,21,22,23,27].

### 4.1. Strengths and Limitations of the Study

This study highlights the role of physiotherapy within PC, addressing a significant gap in the existing literature, as research on this subject in Portugal remains limited. One of its main strengths lies in the use of an appropriate qualitative approach, supported by three FGs that captured the perceptions and experiences of practicing physiotherapists and the challenges they face in local units. This method enabled an in-depth exploration of physiotherapy’s role in promoting comfort across different PC settings: hospital-based, inpatient, and community-based. Qualitative research allows for a deep understanding of participants’ experiences and perceptions, with the flexibly to adapt to different contexts. It is particularly valuable for exploring complex or under-researched phenomena, recognizing individual subjectivity and capturing a diversity of perspectives.

Additionally, it contributes to the generation of knowledge for further investigations. These insights may serve as a foundation for future research and inform the development of standardized policies and procedures to promote the formal integration of physiotherapy into Portugal’s national PC framework, particularly regarding comfort-focused interventions. Among the study’s limitations is the relatively small sample size of 15 participants and the absence of representatives from all districts of mainland Portugal and from Madeira, reaching only the Azores. Future studies should aim to expand the geographic scope to include all regions of the country. This may limit generalizability due to the small sample size, subjectivity in interpretations, and the complexity of data analysis.

Additionally, this research focused exclusively on the perspectives of physiotherapists due to limitations in time, resources, and specific skills. To enrich the understanding of physiotherapy’s contribution to patient comfort in PC, future investigations should incorporate the views of other healthcare professionals, such as physicians and nurses, as well as those of patients and caregivers.

In the healthcare context, qualitative research may be less valued than quantitative approaches, especially when informing clinical guidelines or policy decisions, which constitutes a limitation of this study.

### 4.2. Implications of the Study

This study reinforces the urgent need to strengthen the integration of physiotherapy within PC. It clearly highlights the necessity of establishing national guidelines that formally regulate physiotherapy practice in PC, including specifications for working hours, referral protocols, and integration strategies. Furthermore, increased funding is essential to ensure the availability of specialized physiotherapists in hospitals, inpatient units, and community services. Improving access, particularly by addressing delays caused by late referrals, and promoting equity across both urban and rural settings are also critical steps toward effective service provision.

Regarding the implications for clinical practice, the findings suggest that physiotherapy should be continuously integrated into PC, beginning early in the disease trajectory to prevent complications and preserve functional capacity. Techniques such as lymphatic drainage, respiratory rehabilitation, music therapy, and assisted dance should be more widely adopted. Moreover, empowering caregivers to support patient comfort and tailoring interventions to specific care contexts are essential, prioritizing pain management in hospital settings and mobility enhancement in at-home care.

In the field of education and training, physiotherapy programs must incorporate dedicated content on PC, supported by ongoing professional development and advanced specialization to optimize clinical practice. Interdisciplinary training opportunities involving physiotherapists, physicians, nurses, psychologists, and other professionals are also vital to foster collaboration and enable timely referrals within PC.

Future research should focus on evaluating the impact of physiotherapy on patients’ quality of life, comparing different care models and clinical protocols to establish best practices. In addition, exploring the use of innovative technologies, such as virtual reality, artificial intelligence, assisted robotics, hydrotherapy, aromatherapy, and music therapy, may open new avenues for care. It is also important to investigate how physiotherapists are integrated into broader healthcare teams, identifying both barriers and enablers of effective interdisciplinary collaboration.

## 5. Conclusions

This pioneering qualitative study explored Portuguese physiotherapists’ perceptions of their role in promoting comfort across different PC units. Seven main themes emerged, highlighting the complexity of physiotherapy interventions and the need to adapt to the specificities of patients and care contexts: challenges, diseases, signs and symptoms, family and caregivers, techniques, equipment and materials, and care delivery format.

The findings emphasize the importance of early integration of physiotherapists into specialized teams, with a personalized approach focused on multidimensional symptom management—pain, mobility, dyspnea, function, and well-being—for both oncological and non-oncological conditions. Conventional techniques are applied with adaptations to the PC context and individual tolerance. In community-based units, complementary approaches such as music therapy and spiritual support gain increasing relevance.

Professionals identified challenges, including late integration into teams, lack of specific training, resource shortages, limited recognition of physiotherapy in PC by healthcare professionals and colleagues, and significant regional disparities in access to comfort-focused care.

Despite methodological limitations, the findings provide valuable insights to optimize clinical practice and reinforce strategies to ensure timely access to comfort-focused interventions, potentially serving as an asset nationally and offering contributions to international contexts.

## Figures and Tables

**Table 1:** Themes, subthemes, and corresponding codes.

Themes	Subthemes	Central Aspect of Each Theme
**Challenges**	**Personal barriers**	The theme ‘Challenges’ relates to the barriers faced by professionals. The main barriers include knowledge and training, cultural resistance, underestimation of physiotherapy’s role, poor integration within healthcare teams and services, and the absence of supportive public and financial policies.
	**Organizational** **b** **arriers**	
	**Healthcare system barriers**	
	**Financial and policy barriers**	
**Diseases**	**(a) Oncological** **(b) Non-oncological**	The theme ‘Diseases’ and its subthemes reflect the diversity of conditions treated in PC units, where physiotherapists provide comfort in both oncological and non-oncological pathologies.
**Signs and symptoms**	**Digestive** **Physical**	The theme ‘Signs and symptoms’ and its subthemes encompass the main symptoms requiring intervention in PC units. Physiotherapists contribute to patient comfort by addressing a range of physical, respiratory, psychological, digestive, and other symptoms.
	**Respiratory symptoms**	
	**Psychological**	
	**Physical**	
	**Other**	
**Family and caregivers**	**Collaboration** **Integration** **Requests**	The theme ‘Family and caregivers’ relates to their vital role in physiotherapy within PC units, including collaboration with teams and support in the care process.
**Techniques**	**Pain and mobility management**	The theme ‘Techniques’ refers to physiotherapeutic strategies used to relieve signs and symptoms, maintain function, and promote comfort and well-being.
	**Dyspnea relief and respiratory rehabilitation**	
	**Maintenance of function and autonomy**	
	**Promotion of well-being and overall comfort**	
**Equipment and materials**	**Mobility and** **e** **xercise**	The theme ‘Equipment and materials’ relates to all resources used by physiotherapists in comfort-focused interventions, adapted to the type of technique and intended use.
	**Positioning**	
	**Respiratory** **t** **herapies and** **m** **onitoring**	
	**Electrotherapy and** **t** **hermotherapy**	
	**Complementary** **t** **herapies**	
**Format**	**Setting** **Frequency** **Duration** **Dosage**	The theme ‘Format’ refers to how physiotherapy interventions for comfort are delivered, including the care setting, session frequency, duration, and dosage.

## Data Availability

The raw data supporting the conclusions of this article will be made available by the authors on request.

## Supplementary Materials

The following supporting information can be downloaded at: https://www.mdpi.com/article/10.3390/cancers17132167/s1, Table S1: COREQ checklist 32 items.

## Abbreviations

The following abbreviations are used in this manuscript:

PC	Palliative Care
WHO	World Health Organization
EIHSCP	In-Hospital Palliative Care Support Teams
ECSCP	Community Palliative Care Support Teams
FG	Focus Group

## Appendix A. Interview Guide

**Q1:** Could you clarify in which palliative care settings you usually practice?

**Q2:** Do you consider the non-pharmacological comfort interventions provided by physiotherapy in palliative care to be important?

**Q3:** Could you explain which situations or types of illness, within the scope of palliative care, typically benefit from physiotherapy interventions aimed at comfort?

**Q4:** Based on what was discussed in question 2, could you clarify whether your interventions are more often directed toward oncological or non-oncological conditions?

**Q5:** Among the situations mentioned in questions 2 and 3, which types of illness do you believe benefit most from non-pharmacological comfort interventions delivered by physiotherapists?

**Q6:** Could you describe the non-pharmacological comfort interventions you apply as a physiotherapist working in palliative care?

**Q7:** Which signs and symptoms associated with the previously mentioned conditions benefit most from these non-pharmacological physiotherapy interventions?

**Q8:** In your opinion, are non-pharmacological comfort interventions delivered by physiotherapists directed solely toward patients, or also toward their families and caregivers?

**Q9:** Regarding the non-pharmacological comfort interventions you use most frequently in your palliative care practice (up to three), could you specify the dosage/indication, frequency, duration, and context of application?

**Q10:** For those same interventions (up to three most frequently used), could you describe the materials and/or equipment involved?

**Q11:** Is there anything else you would like to add regarding the topic of this Focus Group that has not yet been discussed?
