# Supplementary material for: Physiotherapy Intervention for Promoting Comfort in Palliative Care Patients: A Focus Group Study
Source: Cancers (Basel). 2025 Jun 27;17(13):2167. doi: 10.3390/cancers17132167 (PMC12249118; doi:10.3390/cancers17132167)
Supplement: Supplementary file 1 [file cancers-17-02167-s001.zip › cancers-3647462-supplementary.pdf]

Supplementary Table S1: COREQ checklist 32 items

**Domain 1: Research team and reflexivity**		
**Personal Characteristics**		
1. Interviewer/facilitator	Which author/s conducted the interview or focus group?	The first author: Célio Cruz
2. Credentials	What were the researcher's credentials? e.g., PhD, MD	The first author: Célio Cruz (Master student)<br>Second author: Ana Querido, Ph.D.<br>Third author: Vanda Pedrosa, Ph.D.
3. Occupation	What was their occupation at the time of the study?	The first author: Célio Cruz (Master student and social worker, Technical Director of Integrated Care Unit)<br>Second author: Ana Querido, Coordinator Professor, Ph.D.<br>Third author: Vanda Pedrosa, Associate Professor, Ph.D.
4. Gender	Was the researcher male or female?	The first author: Célio Cruz (Master student) Male<br>Second author: Ana Querido, Ph.D., Female<br>Third author: Vanda Pedrosa, Ph.D., Female
5. Experience and training What experience or training did the researcher have?<br>Relationship with participants	What experience or training did the researcher have?	The first author: Célio Cruz (Master student), bachelor's degree Investigation, Classes and Seminars from Master in Palliative Care.<br>Second author: Ana Querido, Ph.D., Master and Ph.D. Investigation and Training, supervisor and orient students research in different levels, she´s an integrated member in investigation unit school of Health Sciences.<br>Third Author: Vanda Pedrosa, Ph.D.,<br>Master and Ph.D. Investigation and Training, supervisor and orient students research at different levels, she´s an integrated member in investigation unit school of Health Sciences.
6. Relationship established Was a relationship established prior to study commencement?	Was a relationship established prior to study commencement?	A formal contact with team coordinator was made to allow ethical procedures and authorization for the investigation. After ethical approval, contact was made with all the participants before the interviews, to schedule best time/day/local.
7. Participant knowledge of the interviewer	What did the participants know about the researcher? e.g., personal goals, reasons for doing the research.	Participants have access to the interviewer and meet credentials and goals (personal and academic) for doing the research.<br>They know the credentials from the other two members.<br>They know these credentials within informed consent signed, before the interviews.
8. Interviewer characteristics	What characteristics were reported about the interviewer/facilitator? e.g., Bias, assumptions, reasons and interests in the research topic	The participants know the formal and master´s student interest in the research topic, a master student of Palliative Care with background in Social Work, and Work within Palliative Context for more than ten years.
**Domain 2: Study Design**		
**Theoretical Framework**		
Methodological orientation and Theory	What methodological orientation was stated to underpin the study? e.g. grounded theory, discourse	Our goal was to understand a single phenomenon; therefore, phenomenology served as the main guiding approach for both the study design and participant selection.

	analysis, ethnography, phenomenology, content analysis	
**Participant selection**		
Sampling	How were participants selected? e.g. purposive, convenience, consecutive, snowball	The participants were selected for convenience and access to data, and our goal was to reach the universe of 1 team (we did it).
Method of approach	How were participants approached? e.g. face-to-face, telephone, mail, email	Email and face-to-face
Sample size	How many participants were in the study?	12 participants (the sample is the universe).
Non-participation	How many people refused to participate or dropped out? Reasons?	None, we reach all participants.
**Setting**		
Setting of data collection	Where was the data collected? e.g. home, clinic, workplace	Workplace, in hospital in Palliative Care (inpatient) team.
Presence of non-participants	Was anyone else present besides the participants and researchers?	No one else.
Description of sample	What are the important characteristics of the sample? e.g. demographic data, date	Birthday, years' experience in PC, formation in PC and undergraduate formation.
**Datta Collection**		
Interview guide	Were questions, prompts, guides provided by the authors? Was it pilot tested?	Yes (supplemental material). Yes, 1 pilot tested.
Repeat interviews	Were repeat interviews carried out? If yes, how many?	No, we don´t repeat interviews.
Audio/visual recording	Did the research use audio or visual recording to collect the data?	Only audio for recording to collect the data.
Field notes	Were field notes made during and/or after the interview or focus group?	Yes, during and after.
Duration	What was the duration of the interviews or focus group?	5 to 12 minutes (minimum and maximum).
Data saturation	Was data saturation discussed?	Yes, in the research team and during the data collection, and after, during the transcription.
Transcripts returned	Were transcripts returned to participants for comment and/or correction?	After verbatim transcription, they can comment, correct and validate the transcriptions.
**Domain 3: Analysis and Findings**		
**Data Analysis**		
24. Number of data coders	How many data coders coded the data?	Three data coders. Two along the process and one, always there was a doubt in coding.
25. Description of the coding tree	Did authors provide a description of the coding tree?	Yes, we provide a description and a figure of the coding tree.

26. Derivation of themes	Were themes identified in advance or derived from the data?	We used deductive qualitative research. It allows to use existing theory to examine meanings, processes, and narratives of interpersonal and intrapersonal phenomena.
27. Software	What software, if applicable, was used to manage the data?	We used webQDA to manage and organize the data.
**Reporting**		
28. Participant checking Reporting	Did participants provide feedback on the findings?	Yes, they provide feedback to verbatim and the findings (two experienced participants, with large experience in PC).
Quotations presented	Were participant quotations presented to illustrate the themes / findings? Was each quotation identified? e.g. participant number	Yes, we presented. All quotations are identified, number and profession.
30. Data and findings consistent	Was there consistency between the data presented and the findings?	Yes, we consider consistency between both during writing.
31. Clarity of major themes	Were major themes clearly presented in the findings?	Yes, we defined three major and well-defined themes.
32. Clarity of minor themes	Is there a description of diverse cases or discussion of minor themes?	Yes, when there are minor themes/sub-themes we referred about it. We made a depth description of diverse aspects we found in verbatim.
